# How Motivations for Using Buprenorphine Products Differ From Using Opioid Analgesics: Evidence from an Observational Study of Internet Discussions Among Recreational Users

**DOI:** 10.2196/16038

**Published:** 2020-03-25

**Authors:** Stephen F Butler, Natasha K Oyedele, Taryn Dailey Govoni, Jody L Green

**Affiliations:** 1 Inflexxion Costa Mesa, CA United States; 2 Office of Disease Prevention National Institutes of Health Rockville, MD United States

**Keywords:** buprenorphine-naloxone combination, buprenorphine, motivation, controlled substance diversion, addiction, opioid, opioid medication-assisted treatment

## Abstract

**Background:**

Opioid use disorder (OUD) poses medical and societal concerns. Although most individuals with OUD in the United States are not in drug abuse treatment, buprenorphine is considered a safe and effective OUD treatment, which reduces illicit opioid use, mortality, and other drug-related harms. However, as buprenorphine prescriptions increase, so does evidence of misused, abused, or diverted buprenorphine. Users’ motivations for extratreatment use of buprenorphine (ie, misuse or abuse of one’s own prescription or use of diverted medication) may be different from the motivations involved in analgesic opioid products. Previous research is based on small sample sizes and use surveys, and none directly compare the motivations for using buprenorphine products (ie, tablet or film) with other opioid products having known abuse potential.

**Objective:**

The aim of the study was to describe and compare the motivation-to-use buprenorphine products, including buprenorphine/naloxone (BNX) sublingual film and oxycodone extended-release (ER), as discussed in online forums.

**Methods:**

Web-based posts from 2012 to 2016 were collected from online forums using the Web Informed Services internet monitoring archive. A random sample of posts was coded for motivation to use. These posts were coded into the following motivation categories: (1) use to avoid withdrawal, (2) pain relief, (3) tapering from other drugs, (4) opioid addiction treatment, (5) recreational use (ie, to get high), and (6) other use. Oxycodone ER, an opioid analgesic with known abuse potential, was selected as a comparator.

**Results:**

Among all posts, 0.81% (30,576/3,788,922) discussed motivation to use one of the target products. The examination of query-selected posts revealed significantly greater discussion of buprenorphine products than oxycodone ER (*P*<.001). The posts mentioning buprenorphine products were more likely than oxycodone ER to discuss treatment for OUD, tapering down use, and/or withdrawal management (*P*<.001). Buprenorphine-related posts discussed recreational use (375/1020, 36.76%), although much less often than in oxycodone ER posts (425/508, 83.7%). Despite some differences, the overall pattern of motivation to use was similar for BNX sublingual film and other buprenorphine products.

**Conclusions:**

An analysis of spontaneous, Web-based discussion among recreational substance users who post on online drug forums supports the contention that motivation-to-use patterns associated with buprenorphine products are different from those reported for oxycodone ER. Although the findings presented here are not expected to reflect the actual use of the target products, they may represent the interests and motivations of those posting on the online forums. Buprenorphine-related posts were more likely to discuss treatment for OUD, tapering, and withdrawal management than oxycodone ER. Although the findings are consistent with a purported link between the limited availability of medication-assisted therapies for substance use disorders and use of diverted buprenorphine products for self-treatment, recreational use was a motivation expressed in more than one-third of buprenorphine posts.

## Introduction

### Background

Opioid use disorder (OUD) poses medical and societal concerns, contributing to an increasing economic burden [1]. Approximately 11.8 million individuals older than 12 years misused opioids in 2016, with 11.5 million misusing prescription pain relievers [2]. The same source notes that nearly 2.1 million individuals received past-year specialty treatment for OUD, where only 1 in 5 individuals (21.1%) with OUD received such a treatment. A majority of individuals with OUD in the United States are not enrolled in drug abuse treatment [3]. Nevertheless, the amount of prescribed buprenorphine continues to increase [4-7], as does evidence of use of misused/abused/diverted buprenorphine [8-10]. Previous research suggests that users’ motivations for extra-treatment use of buprenorphine (ie, misuse/abuse of one’s own prescription or use of diverted medication) may be different from motivations to misuse, abuse, or divert analgesic opioid products [3,8,11-14]. All these studies are based on relatively small sample sizes and use surveys, and none directly compare the motivations for using buprenorphine products (ie, tablet or film) with other opioid products having known abuse potential. This study is an effort to use spontaneously occurring Web-based discussion among recreational drug users to better understand the various motivations for use, misuse, and diversion of buprenorphine and empirically examine whether and how the motivations observed for buprenorphine products differ from an opioid analgesic with known abuse potential.

Buprenorphine has been shown to be a safe and effective treatment for OUD, as well as for use in acute detoxification, stabilization, and long-term maintenance of individuals with OUD [15,16]. Opioid maintenance therapy with buprenorphine reduces illicit opioid use, mortality, and other drug-related harms among opioid-dependent individuals [17,18].

Buprenorphine has also been associated with diversion, misuse, and abuse, as the amount of prescribed buprenorphine has increased [4-7]. There is evidence that diversion, misuse, and abuse might vary across buprenorphine products. For example, a recent multi-dataset study [19] found evidence to conclude that prescription-adjusted abuse of the sublingual film was less than the single-entity tablet. Nevertheless, the abuse of buprenorphine quadrupled between 2008 and 2013, when buprenorphine was the fourth most commonly diverted prescription drug in law enforcement cases, behind oxycodone, hydrocodone, and alprazolam [20]. This raises the paradoxical situation: although buprenorphine is intended as a treatment for OUD, it is also likely to be abused, misused, and diverted.

As the prevalence of buprenorphine use outside the context of participation in an authorized, therapeutic program for treatment of OUD increases, evidence is emerging on the differences in the patterns of extra-therapeutic buprenorphine use versus analgesic opioids. Early work by Cicero and colleagues of individuals surveyed in substance abuse treatment [20] found that more than 30% of the individuals reported using buprenorphine to get high, yet only 1.6% of the individuals indicated buprenorphine as their primary drug of choice, compared with 32.4% of the individuals selecting oxycodone as their primary drug, with 29.8% of the individuals selecting heroin. A total of 50% to 60% of those using buprenorphine cited motivations, such as maintenance of abstinence, to aid in weaning off other drugs and manage situations when they needed to function (eg, work or social events). In a more recent survey [8], 52% of the survey respondents reported using buprenorphine to get high, and 4% of the respondents reported it as their drug of choice. In this subsequent survey, 79% of the respondents reported using buprenorphine products to maintain abstinence, and 53% of the respondents reported trying to wean themselves off other drugs. Self-medication for pain (37%) and treatment of emotional problems (19%) were also endorsed by survey respondents as motivations for using buprenorphine. More than 80% of the respondents who used diverted buprenorphine indicated that easier access to a buprenorphine prescriber would increase the likelihood of them procuring a prescription rather than obtaining buprenorphine on their own [8]. These findings are supported by a recent survey of individuals in Rhode Island [3]. This study revealed that the primary motivations underlying the use of diverted buprenorphine were management of withdrawal symptoms and self-treatment of OUD. These authors conclude that restrictive regulations limiting treatment capacity and inaccessibility of existing services have led to diversion of buprenorphine, largely for self-treatment. Other studies have reached similar conclusions [11-14], suggesting the possibility that illicit use of buprenorphine in the United States is motivated, at least for some, by the desire to self-detoxify, self-treat, or manage opioid cravings and other withdrawal symptoms. It is worth noting that in the studies cited, the authors have assumed that the motivations observed with respect to buprenorphine products are different from the motivations for nonmedical use (NMU) of analgesic opioids. Although this is understandable, to our knowledge, this assumption has not been empirically tested.

Discussion on the Web among recreational substance users who post on online drug forums is a method for understanding how drug users express their own motivations for using drugs. Online forums have been considered an ideal medium for individuals who abuse and misuse prescription drugs to communicate with each other [21-23], offering their uncensored ideas and beliefs, discussing trends and preferences, and providing education about recreational drug use [24]. Public online forums can be monitored unobtrusively and may reveal the methods, reasoning, and associated sentiment regarding the misuse of prescription drugs [25,26]. These spontaneous, peer-to-peer discussions also represent a different perspective than obtaining beliefs and practices reported in consented surveys. Discussions regarding prescription opioids on these websites may provide insights into how individuals who post on these online forums view the impetus for use of specific prescription opioid products. Furthermore, the attitudes, preferences, and opinions shared on these online forums can be expected to inform those who view the websites but do not post messages. It is generally believed that most (over 50%) of those who visit online forums are ‘‘lurkers,’’ individuals who frequently read message boards but do not post messages [27]. Thus, discussion about a particular substance or product on these message boards may not only represent the views and interests of those who post messages but also influence the attitudes and interests of the lurkers. Finally, relative to other media sources, such as Twitter, Facebook, or YouTube, online forums appear to retain their relevance on discussions on antisocial topics, such as substance abuse, where anonymity for those who post or read can be maintained.

### Objectives

The aim of this study is to describe the motivations for buprenorphine use, as reported in discussions on the Web. To contextualize the findings of reported motivations and provide a stark contrast, we compared the motivational profile of buprenorphine products with oxycodone extended-release (ER), a nonbuprenorphine, prescription full µ-opioid agonist indicated for analgesia, known to be desirable for euphoric purposes or to get high [28]. Oxycodone ER is consistently reported as highly abused in samples of individuals in chemical dependence treatment [20,29]. In addition, a subanalysis examined for any differences with respect to motivations for using buprenorphine/naloxone (BNX) film as compared with other buprenorphine products. Quantitative and qualitative analytic approaches were used to compare the patterns of motivation-related discussion associated with each product group.

## Methods

### Study Design and Population

This study was a 2-part evaluation comprising (1) retrospective, quantitative analyses of Web-based drug discussion levels of buprenorphine products compared with oxycodone ER and (2) a retrospective, qualitative coding of internet post content regarding the motivation to use these products. A subanalysis was conducted to test for any motivational differences between BNX sublingual film and other buprenorphine formulations.

The study sample was drawn from an archive of internet posts extracted from publicly accessible online forums, which represent a population of recreational substance users and their Web-based communications regarding both illicit and prescription drugs. Posts were identified on 7 online forums that are monitored by National Addictions Vigilance Intervention and Prevention Program (NAVIPPRO’s) Web Informed Services. The forums were chosen based on predefined criteria [25], specifying that the forum must (1) include a message board component; (2) be unedited; (3) promote free discussion of illicit and/or prescription drug use; (4) be open to the public; (5) be privately funded (eg, private donations); (6) be maintained/moderated by volunteers; and (7) be an English-language website (although not all authors who post messages on the Web-form reside in the United States). The posts written between January 1, 2012, and December 31, 2016, were archived in a database for further sampling and analysis. No personal identifiable information related to the author was saved. All research activities conducted for this study were deemed exempt from review by the New England Institutional Review Board.

### Data Sample and Coding

#### Sampling Process for Quantitative Analyses

All the posts referencing a buprenorphine product or oxycodone ER during the study period were collected (product categories are defined in Table 1).

Oxycodone ER was selected to represent a full µ-opioid agonist product with a different medical indication (ie, treatment of pain), which is also known to be desirable for euphoric purposes or to get high [20,28,29]. Product-specific posts were identified from the entire archive of messages posted during the study period using standardized queries to identify posts that contained text matching search-string criteria. Search-string criteria for products included common misspellings, slang, and/or wildcard characters (eg, suboxone%, _xone, sub%, and bupe%). Search-string criteria were also generated to capture possible motivation-related discussion following a review by the research team of the literature and a manual review of a sample (approximately 500) of buprenorphine and oxycodone ER posts. In addition, consensus criteria were generated (eg, therapy%, detox%, rehab%, sober%, quit%, abuse%, rush%, high%, euphor%, nod%, relax%, and buzz%). These criteria were used to identify relevant query-selected posts, along with exclusion terms, to minimize the number of posts that did not pertain to the specified product or contain motivation-related discussion. Note that multiple posts may be submitted by the same author and multiple motivations may be mentioned by the same author in a single post or across multiple posts. In addition, more than one of the target products may be mentioned in a single post. The posts mentioning BNX sublingual film were classified as such and excluded from the other buprenorphine product categories. The posts that mentioned a buprenorphine product and oxycodone ER were included in both categories, so these categories are not mutually exclusive.

**Table 1:** Query inclusion terms.

Product	Inclusion terms
Buprenorphine/naloxone film^a^	Suboxone film; associated slang and common misspellings
Other buprenorphine products	Subutex; Zubsolv; Bunavail; Suboxone tablets; generic buprenorphine/naloxone and single-ingredient buprenorphine tablets; and associated slang and common misspellings
Any buprenorphine product	Buprenorphine/naloxone (film or other buprenorphine products)
Oxycodone extended-release^b^	Original OxyContin extended-release; reformulated OxyContin extended-release; oxycodone extended-release; and associated slang and common misspellings

^a^Posts containing specific mention of buprenorphine/naloxone sublingual film were classified in this category even if the posts also included a discussion of other buprenorphine products.

^b^It is possible for a post to mention a buprenorphine product and oxycodone extended-release. In those cases, the post would be captured in both categories, so there may be some level of overlap with the buprenorphine category.

### Analytic Methods for Quantitative Analyses

Percentages and 95% CIs of posts (ie, number of motivation-to-use posts per total posts in archive×100) and authors (ie, number of motivation-to-use authors per total authors in archive×100) were included for each product category. Analyses compared the extent to which motivation was discussed and the number of people discussing motivation of the target products relative to the total discussion/authors in the Web Informed Services archive.

#### Sampling Process and Sample Size Calculations for Qualitative Evaluation

**Figure 1:**
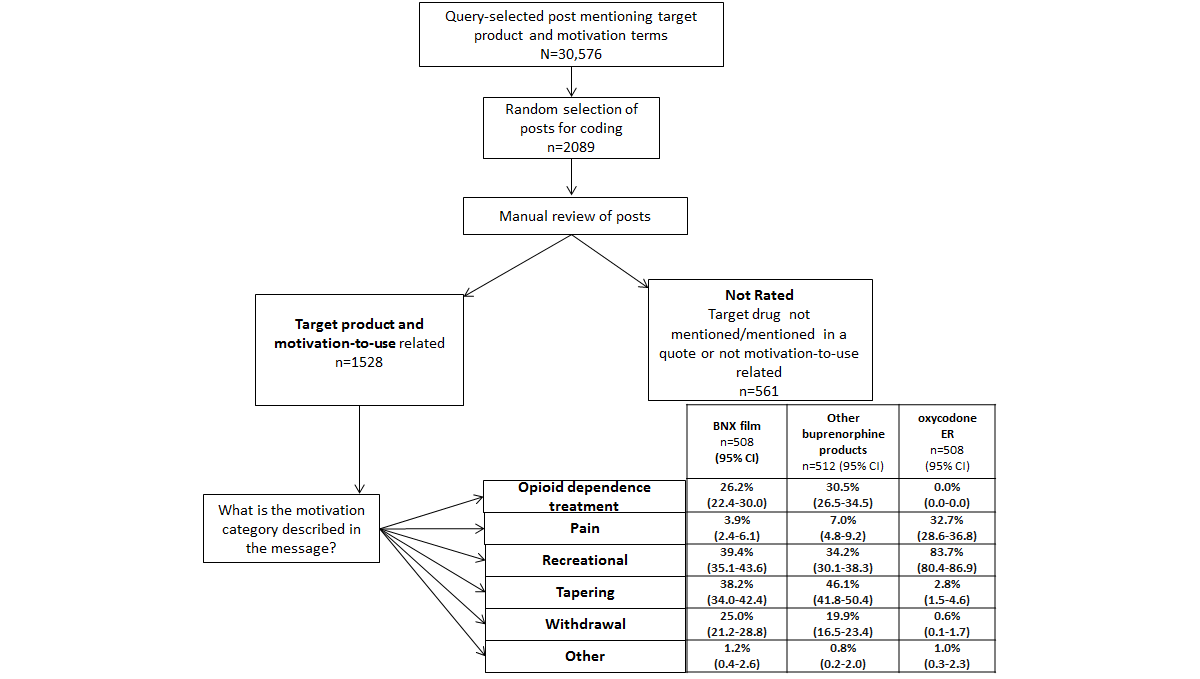
Motivation-to-use content analysis flow chart. BNX: buprenorphine/naloxone; ER: extended-release.

The posts to be analyzed for motivation-to-use comparisons were selected from the pool of query-selected posts as described above. Power analyses required 500 posts per prescription opioid category. To have a sufficient sample size for the subanalysis to examine differences between BNX sublingual film and other buprenorphine products, N=1500 was proposed to ensure 100 posts across each year of the 5-year study period for BNX sublingual film, other buprenorphine, and oxycodone ER. From among the pool of query-selected messages discussing the motivation to use the target products, posts were randomly selected for the evaluation of the motivation-to-use analyses. As some posts may not meet the inclusion criteria, a total of 2089 posts were sampled and manually reviewed to ensure that all posts coded pertained to the specified product and contained motivation-related content (see the flowchart in Figure 1). The primary analyses compared any buprenorphine and oxycodone ER. The category of any buprenorphine was created by combining the BNX sublingual film and other buprenorphine categories (Table 1).

#### Content Analysis and Qualitative Evaluation of Motivation

A formal content analysis of the motivation to use was conducted on the random sample of posts related to any buprenorphine (BNX sublingual film and other buprenorphine) and oxycodone ER. Motivation to use was defined as any post discussing the rationale behind the use of a prescription opioid compound, including use as prescribed. The posts were reviewed by 2 trained coders. Each post was first categorized by coders as having content that was motivation related or not motivation related and relevant to one of the target products (Figure 1). The posts that were determined to contain motivation-to-use discussion were further coded into 6 categories: use to avoid withdrawal symptoms, use for pain relief, use to taper from other drugs, use to treat OUD, use for recreational purposes, and/or other motivations (Table 2). The posts that were determined not to pertain to the target drug of interest or have any motivation-related content were omitted from coding. Thus, posts were sampled, reviewed, and then resampled to ensure that the number of posts in each category was consistent with the power analysis requirements.

**Table 2:** Motivation-to-use category definitions and examples used to code motivation of use.

Motivation-to-use category^a^	Definition and examples
Opioid use disorder treatment	Pertains to any post that discusses the use of a product for opioid use disorder treatment or maintenance, using only products prescribed by a medical professional. For example, I was prescribed product X to get off product Y; My doctor gave me product X to help me get clean.
Pain	Posts that discuss the use of a product to treat physical pain. The source of the product is not considered within the context of this category; only the fact that it was discussed as being taken to mitigate pain is considered. For example, Product X is strong enough to alleviate pain symptoms; I was surprised that product X helped with my chronic pain.
Recreational	Posts that reference the recreational use of a product, including references to getting high, obtaining enjoyable sensations, and using for general enjoyment. For example, This is my first-time using product X to get high; I took product X to feel euphoric.
Tapering	Posts that discuss the use of a product to reduce or eliminate the use of another product. This includes self-medication. For example, If you want to taper down, you might consider taking product X; Product X helped me reduce my use of product Y.
Withdrawal	Pertains to posts that discuss the use of a product to mitigate or treat opioid withdrawal symptoms. For example, I use product Y to treat withdrawal symptoms; I need to wait until withdrawal symptoms start before using product Y.
Other	Pertains to posts that contain references to use a product for a purpose not described in the other motivation categories (eg, as they could not afford another prescription opioid product or to self-medicate depression). For example, I take product X to help with depression and anxiety; I regularly use product Y, but I did not have the money and restored to using product X.

^a^Motivation-to-use categories are not mutually exclusive, a single post may contain more than one motivation.

### Analytic Methods

#### Intercoder Agreement

To assess the reliability of the coding, a random subsample of at least 20% of all posts was coded by both the primary and secondary coder, with the remaining posts coded by the primary coder [25]. The posts were assigned to the primary or secondary coder by a random-number generator. The coders were unaware of the posts coded by the other coder. For the overlapping sample, intercoder agreement was assessed. When coders disagreed, a consensus decision achieved a single set of codes for analysis. Intercoder agreement was calculated using the Kappa statistic [30]. Reliability was separately calculated for 2 buprenorphine categories (BNX sublingual film and other buprenorphine), along with oxycodone ER. Acceptable intercoder Kappa values were obtained for 2 coders across motivations and products, with an overall Kappa of κ=0.85 (Kappa ranged from κ=0.77 to κ=0.91), suggesting excellent agreement.

#### An Analytic Approach Toward Qualitative Post Analyses

Comparisons of the types of motivation discussed were calculated as percent and CIs (motivation-to-use category divided by the total sample randomly chosen to be coded). Comparisons of percents and percentages across product categories utilized the Chi-square statistic. The Type I error was set at alpha=.05. These comparisons were intended to compare the types of motivations discussed for the target products for the primary analyses (any buprenorphine versus oxycodone ER) and between BNX sublingual film and other buprenorphine products.

## Results

### Data Evaluation

A total of 3,788,922 posts were collected on the Web on all topics between January 1, 2012, and December 31, 2016, on the monitored online forums. Among all posts, 1,393,059 query-selected messages contained motivation-to-use–related mentions by a total of 67,156 unique authors (ie, posts submitted by the same username). Of the 3,788,922 motivation-related posts, 30,576 posts by 10,889 unique authors (some people authored multiple posts) contained a query-identified reference to one of the target product categories and motivation-related term(s)—Table 3.

**Table 3:** Post and author counts of evaluated product categories (between January 1, 2012, and December 31, 2016).

Evaluated categories	Post counts (N=3,788,922)		Unique author counts (N=84,711)	
	Frequency, n (%)	95% CI	Frequency, n (%)	95% CI
Posts discussing motivation-to-use target product categories	30,576 (0.81)	0.80-0.82	10,889 (12.86)	12.63-13.08
Any buprenorphine product	18,170 (0.48)	0.47-0.49	6337 (7.48)	7.30-7.66
Buprenorphine/naloxone sublingual film^a^	3522 (0.09)	0.09-0.10	1772 (2.09)	2.00-2.19
Other buprenorphine products	14,648 (0.39)	0.38-0.39	4565 (5.39)	5.24-5.68
Oxycodone extended-release	12,406 (0.33)	0.32-0.33	4552 (5.37)	5.22-5.53
Total posts including motivation key words	1,393,059 (36.77)	36.72-36.82	67,159 (79.28)	79.01-79.55

^a^Posts containing specific mention of buprenorphine/naloxone sublingual film were classified in this category, even if the posts also included a discussion of other buprenorphine products. It is possible for a post to mention a buprenorphine product and oxycodone extended-release. In those cases, the post would be captured in both categories, so there may be some level of overlap.

### Quantitative Evaluation of Online Forum Discussion

Estimates of the level of drug motivation-to-use discussion relative to all discussions on these online forums and 95% CIs derived from percents of target posts/total archive per 100 posts for each product category are presented in Table 3. The primary analysis of any buprenorphine versus oxycodone ER product revealed a significantly greater level of discussion (ie, mentions of the product, along with at least one motivation keyword) regarding any buprenorphine product (18,170/3,788,922, 0.49%) than oxycodone ER (12,406/3,788,922, 0.33%; *P*<.001). Similarly, buprenorphine was discussed by more authors (6337/84,711, 7.50%), compared with oxycodone ER (4552/84,711, 5.44%; *P*<.001; Table 3).

Within buprenorphine products, significantly fewer posts discussed motivation to use BNX sublingual film (3522/3,788,922, 0.09%) than other buprenorphine products (14,648/3,788,922, 0.40%; *P*<.001; Table 3). Table 3 also shows that BNX sublingual film had fewer authors (1772/84,711, 2.18%) than the other buprenorphine product group (4565/84,711, 5.4%; *P*<.001).

### Qualitative Evaluation of Motivations for Use and Discussion Themes

The primary comparison of interest was motivation to use any buprenorphine product versus motivations discussed in posts referencing oxycodone ER. As can be seen in Table 4, the pattern of references for motivation to use buprenorphine products was very different from oxycodone ER.

Every coded motivation category, except other, was significantly different for these 2 product categories (*P*<.001; Table 4). Unsurprisingly, the motivations coded for buprenorphine posts reflecting buprenorphine use in a way that is consistent with self-medication aims, such as tapering (430/1020, 42.20%), managing withdrawal (230/1020, 22.50%), and opioid dependent treatment references (289/1020, 28.30%), were much more likely to be observed than in posts referencing oxycodone ER. Oxycodone ER posts discussed these self-medication–related motivations in 0 posts for OUD treatment to 14 out of the 508 coded posts (2.8%; *P*<.001 for all comparisons except for the other category, which was not significant; Table 4).

Discussion related to the use of buprenorphine products for OUD treatment largely mentioned procuring the product via a prescription from a medical professional. References included use as prescribed through current participation in a maintenance program or past participation in a treatment program, which could no longer be afforded. Nearly 25% of the posts were coded for both OUD treatment and tapering motivations, particularly when there was mention of past participation in an OUD treatment program. However, discussions of current use tended to be associated with discussions of self-detoxification (ie, managing withdrawal or tapering from other drugs).

Recreational use, on the other hand, was much more likely to be mentioned in oxycodone ER posts (425 out of 508 posts, 83.7%) than in buprenorphine posts (375 out of 1020 posts, 36.76%; *P*<.001). However, it should be noted that this suggests that more than one-third of buprenorphine posts mentioned use to get high, second only to tapering (430/1020 or 42.20%). An informal content review of posts referencing buprenorphine products’ (BNX sublingual film and other buprenorphine products) recreational use suggests a wide range of subtopics, including seeking or obtaining feelings of euphoria and experiencing hallucinations and sickness. Recreational use posts also discussed the ease of accessibility and difficulties while abusing buprenorphine products formulated with naloxone. Several recreation-related buprenorphine posts referenced use by alternative routes of administration, including intravenous (n=54), intranasal (n=23), rectal (n=6), and smoking (n=3; see Multimedia Appendix 1 for some examples).

The use of oxycodone ER to treat past and/or present physical pain accounted for nearly one-third of the Web-based discussion of this product compared with less than 5.50% (56/1020) for buprenorphine (Table 4). There were several mentions of use for both pain relief and recreational use, where an individual could be prescribed oxycodone ER for pain management but could also subsequently progress to recreational use over the course of therapy (see Multimedia Appendix 1). The use of buprenorphine products for pain was infrequently discussed (56/1020, 5.50%) and, when discussed, generally reflected the authors’ unfamiliarity of their use for pain management. Other unspecified motivation to use buprenorphine was rarely coded (10/1020, 1.00%) and included off-label use of the product to treat depression and social anxiety.

We also compared the discussion specific to BNX sublingual film with other buprenorphine products. As can be seen in Table 5, the motivation to use other buprenorphine products for tapering (236/512, 46.1%) was significantly greater than for BNX sublingual film (194/508, 38.2%; *P*=.01; Table 5). The use of other buprenorphine products to treat physical pain (36/512, 7.0%) was also significantly different from BNX sublingual film (20/508, 3.9%; *P*=.04). Discussion of OUD treatment, recreational use, withdrawal, and other topics for other buprenorphine products were not significantly different from sublingual BNX sublingual film (Table 5).

**Table 4:** Percentage of posts mentioning specific motivation-to-use categories and Chi-square *P* values for pairwise differences.

Motivation-to-use category	Buprenorphine products (N=1020)^a,b^		Oxycodone extended-release (N=508)^a,b^		Buprenorphine versus oxycodone extended-release
	Frequency, n (%)	95% CI	Frequency, n (%)	95% CI	*P* value^c^
Opioid use disorder treatment	289 (28.30)	25.6-31.1	0 (0.0)	0.0-0.0	*<.001*
Pain	56 (5.50)	4.1-6.9	166 (32.7)	28.6-36.8	*<.001*
Recreational	375 (36.80)	33.8-39.7	425 (83.7)	80.5-86.9	*<.001*
Tapering	430 (42.20)	39.1-45.2	14 (2.8)	1.4-4.2	*<.001*
Withdrawal	230 (22.50)	19.9-25.0	3 (0.6)	0.1-1.9	*<.001*
Other	10 (1.00)	0.4-1.6	5 (1.0)	0.3-2.3	.99

^a^Number of posts coded for motivation content for each product category.

^b^As posts may mention more than one motivation-to-use, percentages do not add up to 100%.

^c^*P* values in italics are significant.

**Table 5:** Percentage of posts mentioning specific motivation-to-use categories and Chi-square *P* values for pairwise differences in buprenorphine/naloxone sublingual film versus other buprenorphine products.

Motivation-to-use category	Buprenorphine/naloxone sublingual film (N=508)^a,b^		Other buprenorphine products (N=512)^a,b^		Sublingual film versus other buprenorphine
	Frequency, n (%)	95% CI	Frequency, n (%)	95% CI	*P* value^c^
Opioid use disorder treatment	133 (26.2)	22.4-30.0	156 (30.5)	26.5-34.5	.14
Pain	20 (3.9)	2.4-6.1	36 (7.0)	4.8-9.2	*.04*
Recreational	200 (39.4)	35.1-43.6	175 (34.2)	30.1-38.3	.09
Tapering	194 (38.2)	34.0-42.4	236 (46.1)	41.8-50.4	*.01*
Withdrawal	127 (25.0)	21.2-28.8	102 (19.9)	16.5-23.4	.06
Other	6 (1.2)	0.4-2.6	4 (0.8)	0.2-2.0	.55

^a^Number of posts coded for motivation content for each product category.

^b^As posts may mention more than one motivation-to-use, percentages do not add to 100%.

^c^*P* values in italics are significant.

## Discussion

### Principal Findings

This study compared the motivations to use expressed by recreational substance users in Web-based posts for buprenorphine products and an opioid analgesic product with known abuse potential (oxycodone ER). As expected, based on previous analyses of Web-based discussions of oxycodone ER use for recreational purposes [25,26], motivations to use oxycodone ER were primarily related to recreational use and treating pain (the labeled indication). It is unsurprising to note that on an online forum dedicated to recreational use of substances, recreational use of oxycodone ER (83.7%) was the most frequently coded category for this medication, with the second most often coded motivation being pain treatment (32.7%). In contrast, although recreational use of buprenorphine products was observed, at 36.8%, it was coded much less often than oxycodone ER–related posts.

The finding that motivation-to-use patterns of buprenorphine are different from a prescription opioid indicated for the treatment of pain is consistent with other studies [11-14] using different data sources and populations. However, to our knowledge, this study is the first to directly compare motivation to use oxycodone ER with buprenorphine products. This direct comparison confirms the notion that buprenorphine products are discussed differently than oxycodone ER by those who post messages on online forums dedicated to recreational substance use. Although it would have been interesting to examine additional analgesics, the intensive work involved in coding required that we identify a single reasonable representative of an opioid analgesic with known abuse potential, in this case, oxycodone ER. The study period (2012 to 2016) was well after the 2010 reformulation of oxycodone ER, although it is possible that some oxycodone ER discussion involved references to the prereformulation version. Although not tested directly, it may be reasonable to speculate that the motivation-to-use pattern observed for oxycodone ER would be similar to other full µ-opioid agonists. Consider, for instance, a study by McNaughton and colleagues [25], who coded posts from the Web Informed Services archive for the extent to which various opioid compounds were endorsed for recreational use; they found endorsement for abuse to be greatest for oxymorphone, followed by hydromorphone, hydrocodone, oxycodone ER, morphine ER, and tramadol. Oxycodone ER was in the middle of this group of products and was significantly less endorsed for abuse than oxymorphone and hydromorphone, and it was significantly more endorsed than tramadol. The calculated endorsement ratio for oxycodone ER was not significantly different from hydrocodone or morphine ER. Thus, one might expect that, with the exception of tramadol, the other compounds’ posts would be similarly discussed in a recreational context on the Web.

Furthermore, although some differences were observed in this study between BNX sublingual buprenorphine and other buprenorphine products, the overall pattern of motivations examined was quite similar. The examination of both posts referencing BNX sublingual film and posts referencing other buprenorphine products revealed a range of motivations related to addiction management, including OUD treatment, and self-management of tapering and withdrawal. Although some interest in pain relief was detected, this tended to be at much lower levels than the discussion of efforts to quit or manage opioid withdrawal.

Despite the clearly articulated interest in the use of buprenorphine products for withdrawal management and self-tapering, the recreational use of BNX sublingual film and other buprenorphine products was discussed just as frequently as the use of these products for addiction management, underscoring the dual use of these products for both recreational and self-medication intent. Therefore, self-medication in this context does not necessarily imply that the aims of the user is to decrease or stop using opioids. Furthermore, the way the authors discuss recreational substance use of buprenorphine products (BNX sublingual film or other buprenorphine) may be different from the way products such as oxycodone ER are discussed. The presence of naloxone, as well as the film or sublingual tablet formulations, may impact the overall sentiment expressed in Web-based posts regarding recreational use, which have been shown to be different for different products [25,31]. Further studies are required to investigate whether the nature of recreational-use discussions of buprenorphine differ from recreational-use discussions about opioid analgesics.

Owing to the unstructured nature of the online forum content, the source of procurement could not be reliably determined. It is possible that a lack of reference to obtaining a buprenorphine product as a part of an addiction treatment program potentially involved diverted buprenorphine products. It is also possible that some references to tapering and withdrawal in these posts may be related to appropriate OUD treatment. On the basis of post content, it was not always possible to distinguish appropriate medically supervised treatment from the use of diverted product to self-medicate. Nevertheless, this study’s findings are consistent with studies specifically investigating diverted buprenorphine use [3,8]. These authors and others [32] suggest that health insurance coverage, limited Medicaid coverage, and stigma against pharmacotherapy for OUD have resulted in a shortage of treatment capacity and led to inaccessibility of existing services. Consequently, the persistence of these societal conditions is likely to ensure that the individuals in need of treatment will continue to self-treat with diverted medications. Although we concur, generally, with this conclusion, recreational use (ie, use to get high) was cited relatively frequently in the coded posts—a finding consistent with other studies [8]. It may be a mistake to assume that legitimate access alone accounts for buprenorphine use outside of a treatment program.

### Limitations

This study has limitations that should be considered. A common concern with respect to data collected from online forums is that those who post may not be truthful. Although the veracity of any individual post cannot be ascertained, it should be noted that individuals who participate in the examined forums represent stable communities of drug users who are self-policing; therefore, the posted information that is inconsistent with others’ experiences tends to be corrected by the online community [33]. As with any self-report data, self-report biases cannot be ruled out. However, the anonymity that is inherent on these forums, as well as the fact that the opinions expressed are targeted to peers and not researchers or other authorities, renders self-report bias in a different light for these data.

Although the online forums included were selected according to a priori criteria, they were not randomly selected. Sampling bias may exist in trends discussed and users’ traits of selected forums versus unsampled forums; however, the included forums were selected based on the volume of recreational-use discussion, making it a saturated sample. The forums used in this study may differ in the amount and tone of discussion devoted to using and potentially abusing pharmacological products. This study’s findings may only be reflective of communities of recreational drug users who participate in online forums and may not be representative of all Web-based discussion. In addition, although discussions on the Web may capture the interests, intentions, and motivations expressed by those who post on the Web, these data are not intended to capture the actual use of the target products.

We have noted that the examination of post content from online forums provides many advantages for the researcher, including the ability to eavesdrop on conversations among individuals who use drugs illicitly rather than obtain information through some authority (ie, researchers, law enforcement, and health care workers). However, a disadvantage of the method is that the anonymity prevents us from being able to characterize who the authors are and place them within the context of known populations of illicit and NMU of substances. A study [34] attempted to characterize visitors to a single, large online forum, Bluelight.org, using a survey. Most (63%) of the respondents from a sample 897 respondents were from the United States; the remaining 37% of the respondents were from the United Kingdom (12%), Australia (9%), Canada (6%), and 11% were from *other*. The respondents had an average age of 25 years (SD 12) and were mostly male (76%) and white (86%), and almost 80% of them had some college education, graduated college, or had postgraduate training. About 35% of the respondents reported some alcohol or drug treatment and 31% of the respondents reported past 30-day NMU of a prescription opioid. To place these demographics into context, we compared them with recent NAVIPPRO Addiction Severity Index-Multimedia Version substance use treatment center data [35]. Compared with the demographics of the internet responders cited above, fewer of the 217,240 treatment patients were male (65%) and white (60%), and 22% of these patients reported past 30-day prescription opioid NMU, compared with 31% of the online forum respondents. Another NAVIPPRO treatment center study [36] of prescription opioid NMU reported on education level and found 30% of the patients with some college or higher level of education and an older population (nearly 80% of them were older than 24 years). Although the inability to precisely describe the population of authors in this study remains a limitation, it seems likely that the present sample is younger and more well educated than the individuals in treatment for substance use disorder.

We acknowledge that the selection of the specific query terms used to identify posts discussing the target products and potential motivations may have excluded terms that omitted relevant posts to an unknown extent. Furthermore, differentiating among the motivation categories presented in Table 2 requires some interpretation of motives. Tapering or managing withdrawal symptoms does not imply a desire on the part of the author to stop using drugs or seek treatment. However, the high intercoder reliability obtained while coding these categories, as well as the clear differentiation between the results for buprenorphine and oxycodone ER, suggests that the findings presented here are reliable and valid.

Only a sample of posts was selected from the 5-year study period, and longitudinal motivations for use trends were not analyzed. The identification of product-specific posts by querying based on keywords is incomplete; it may conflate some posts discussing more than one of the target products and may have missed some motivations that were not captured in the keyword list. Furthermore, as querying methods capture posts that are determined to be irrelevant to the target topic upon review by trained coders (in this case, specific product mentions and discussion of motivation-to-use), the quantitative analyses based on querying results may overestimate the amount of the Web-based discussion presented here. However, it is unlikely that this lack of precision differentially impacts the products compared, as human review of the sampled posts resulted in almost identical proportions of excluded posts for the products examined. It is also possible that there are terms and slang that are unknown to us or references to a product or motivation that were not captured in this study. However, coders spend considerable time following threads and discussions on the online forum and becoming familiar with the unique communication styles of these communities on the Web. Therefore, it is likely that coders for this study were able to capture the essence of the meaning available to the majority of forum visitors [31].

The posts analyzed here were posted over several years, ending in 2016. It is acknowledged that much has changed since then. In recent years, the use of illicit fentanyl has increased dramatically [37], although at the same time, the prescriptions dispensed for opioid analgesics have decreased, largely as a response to Centers for Disease Control and Prevention guidelines published in 2016 [38,39]. The recent introduction of buprenorphine subcutaneous formulations [40] may further impact the Web-based discussion of buprenorphine products. Future investigations should examine how these changes are reflected in the Web-based discussion of opioids in general, particularly buprenorphine.

### Strengths

The study strengths include the use of a relatively large sample size, inclusion of quantitative and qualitative analyses, and the use of systematic and consistent methods that build on previously published studies. Additional strengths include the use of a standardized coding methodology, analysis of Web-based post discussions with acceptable interrater agreement, and the systematic archiving and storage of forum posts over time, allowing for retrospective evaluation of data and circumventing bias of forum moderators who may delete older posts.

### Conclusions

Although prior studies have suggested that the motivation to use diverted buprenorphine products is different from the motivations for abuse of opioid analgesics [20,28,29], none have directly compared motivations for abuse of these products. In this study, we directly compared motivations for using or abusing buprenorphine products with those expressed for one, widely abused prescription opioid indicated for analgesia and known to be desirable for euphoric purposes or to get high (ie, oxycodone ER). Compared with oxycodone ER, discussion of buprenorphine was significantly more likely to reflect OUD treatment, tapering, and withdrawal management. Buprenorphine products were associated with less discussion of use for recreational purposes or pain relief relative to oxycodone ER. These findings are consistent with the work of others. Some authors have suggested a link between the limited availability of medication-assisted therapies and use of diverted buprenorphine products [3,8,32]. However, this study and others [4-7] found evidence for a meaningful level of misuse, abuse, and diversion of the product, which may or may not be associated with the availability of medication-assisted therapy. We observed little difference in motivation-to-use patterns between BNX sublingual film and other buprenorphine products. Finally, this study also supports the value of Web-based discussions among a population of interest, namely, recreational users of drugs, to better understand motivations for using different prescription opioid products.

## Appendix

Multimedia Appendix 1 Examples of motivation-to-use internet discussion posts.

## Abbreviations

BNX: buprenorphine/naloxone
ER: extended-release
IBH: Integrated Behavioral Health
NAVIPPRO: National Addictions Vigilance Intervention and Prevention Program
NMU: nonmedical use
OUD: opioid use disorder
PLC: public limited company
