# Supplementary material for: How Motivations for Using Buprenorphine Products Differ From Using Opioid Analgesics: Evidence from an Observational Study of Internet Discussions Among Recreational Users
Source: JMIR Public Health Surveill. 2020 Mar 25;6(1):e16038. doi: 10.2196/16038 (PMC7142742; doi:10.2196/16038)
Supplement: Multimedia Appendix 1 [file publichealth_v6i1e16038_app1.docx]

| Motivation-to-use category | Examples of buprenorphine/naloxone sublingual film posts | Examples of Other buprenorphine products posts | Examples of oxycodone extended-release posts |
| --- | --- | --- | --- |
| Recreational | “The strips are unabusable, no method of iv use has even been reported. Smoking Suboxone in the strip will leave you with a mouthful of binders as well as some seriously degraded Suboxone.”; “last night I got 8mg of Suboxone and it was the strip that you put under your tongue. I took the whole strip and it tasted awful, I almost spit it out…. My head started feeling heavy, my legs got tingly, this giant wave of euphoria took over my body. I could hardly keep my eyes open because I was so relaxed. It was the best high I have ever experienced.”; “injected about 2mg Suboxone about 20 mins ago... very familiar with IV and curious about subs. Just want anyone, who cares to know, that its possible to successfully enjoy IVing the films.”; “I have developed a good tek for snorting suboxone strips: Place the piece in a small container, add 2-3 drops of visine or clear eyes to the surface of the Suboxone piece. Then, add about 0.5ml of water, stir it up with a piece of something, then sniff it up each nostril. The visine or clear eyes tends to acidify the buprenorphine base that's in the Suboxone, making it more water-soluble”; “Smoked suboxone for the first time tonight. Put a piece of a strip on foil and inhaled. I've been on and off subs for years and this is the first time I actually got an opiate high from Suboxone. Tastes just like OC's too!” | “I just quit IV'ing Subutex, even though the shots were completely clean (or so I thought), it was making my kidneys swell up. No blood in the urine or anything like that, but pain around the kidney area. Going back to sniffing/sublingual”;  “I ended up getting 3 or 4 Subutex 8 MG and i went ahead and took a couple k-pins, prepared a 2mg solution of subutex mixed with warm water and when I shot it and BAM pure buprenorphine rush.” | “First tried Oxy circa 2003 in college. Didn't know what I was getting myself into. Up to that point I was a responsible drug user / typical college kid, just smoking herb / drinking / tripping&amp; raving on occasional weekends. Progressed from a relaxing Satuday with a 20mg to using throughout the week, up to 40s and 80s. Driving 6 hours round-trip for pills. Went from eating to snorting; Tried to shoot a pill once, thankfully failed (a line I swore i'd never cross)”; “just gone crazy and smoked a whole 80 mg OC in 4 quarters JUST WANTED TO FEEL THE OC HIGH AGAIN and I'm so stressed about uni. Then snorted 2 more... STill not amazing xanax makes the high less good. and tolerance. oh well of to sleep i go.” |
| Tapering and withdrawal | “Let me 1st say I use it with the sublingual strips which I find to be MUCH easier to cut up into precise 1mg and sub-1mg doses. I use a square cut out of .5-1mg doses, 1x sometimes 2x/day....But I've been able to taper down to a .5-1mg dose per day, something that I thought wouldn't be possible for me.”; “I have used strips before but more on the abusive side of the fence rather than what they are prescribed for and feel the w/ds are worse than opiates. How long (hours) after last bump of H should I wait before starting the strip taper process? And if done correctly how intense do u expect the ending w/d's to be?”; “if you can get your hands on 1-2 strips of Suboxone it will significantly help you through the acute stage of your withdrawal.” | “still doing good – am really quite surprised how much of the craving for heroin has gone since on the Subutex- anyone else found this?”; “I started Suboxone3 yrs ago and switched to Subutex the last year, weaned down to 1-2mg/daily. I stopped cold turkey 12 days ago and it's been quite a roller coaster, but I am definitely glad I chose to do it.”; “Subutex will still cause participated withdrawals, as buprenorphine is a competitive agonist (actually more powerful of one than naloxone/Narcan).” | “i was uncomfortable but i managed through a few days of this until i decided to use oxycontin to help me taper my dosage down. thank god for the OP formula so i did not bother to abuse it. this is a perfect oppertunity for me to kick as i am going on a plane trip next week . . .”; “What you need to do is slowly taper your dose down, . . The problem is that you're in the United States, so you have the s**ty form of OxyContin, so the tablets are a real bitch to split. My heart goes out to you, tapering off OCs used to be so much easier until they reformulated.” |
| Opioid use disorder treatment | “Current use; Up to 1.5g H daily, or 120-150 Roxi. All snorted. Have 30 x 8mg total Subs for now. Another mtng w Dr on Mon to see how I am doing, then I guess another 90 Suboxone will be prescribed.”; “I am on a suboxone program and if I choose to spare a few films to help someone out that has no means of getting on them right now, it is at my discretion to do so. I have been clean for 2 and 1/2 years and was clean for a year and 8 mths before I ever knew anything about Suboxone.”; “I went to detox in Jan 2012. When I got out, I did outpatient and then started on suboxone maintenance group….So I started smoking pot again, which my suboxone group was VERY against. So, after the last month of marijauana use, my car **** the bed so I could no longer attend the group meetings, they gave me a choice to return to outpatient for a few weeks, then come back to the group, or to do a 2 week taper.” | “I used Subutex many many years ago when it first came out and imo [in my opionion] that one is the best…I felt great while taking that…I stayed clean after my first detox”; “He recently tried changing onto subutex but it made him feel too high kinda like he was on uppers. The anxiety also made him want to take valium all the time which is something he dont wanna do.Does anyone have any advice??will this be a bit more sedating than subutex is??” | No posts were discussed in this study |
| Pain | “Why did I need Suboxone? Severe lower back pain L5/L6 disc area is bad, facet joints are bruised due to an accident back in 2010. Why am I being prescribed Suboxone as a pain management drug?”; “I do not understand why you are prescribed Suboxone for chronic pain. Buprenorphine can be good for pain management but usually not to someone who is already tolerant on opioids.” | “The idea of using buprenorphine for chronic pain at dosages above 1-2mg/day is ludicrous IMO. Subutex, like Suboxone was formulated to treat recovering opiate addicts, not for pain management. Like it was already mentioned, bupe can only be effective for pain control in microgram dosages, which is why the bupe formulas intended for pain management” | “However I would suggest there may be a better way of accomplishing what you want and using the pain meds as intended and prescribed. Right now I do not have cancer but have chronic pain. I am currently prescribed Oxycontin 60mg 3 times a day as my long acting pain med.”; “I went down the opiate path after crushing the soft tissue between the vertebra in my spine and being prescribed high dose (60mg) pure codeine pills with a pretty much unlimited script. After 3 years of that I was then put on oxycontine and then morphine patches, after a year of that I had a real problem that had nothing to do with back pain but everything to do with opiate addiction/dependence.” |
| Other | “Even if your doctor is using Suboxone off-label for depression and claims you need to be on it for eternity, why the **** would he start you off at the absolute highest amount that can legally be prescribed? Wouldn't he want to find the lowest effective dose and go from there?” | “I can tell u the REAL best drugs for depression/anxiety. the ones that have worked for me. bupe is suppose t work great. im only trying it today for the second time” | “I was just thinking about this topic this morning, before I saw the post. If I could maintain the rest of my life on oxycontin, I probably would. It makes me feel motivated during the day, and when combined with some weed, allows me to crash out at night without tossing and turning for hours. Definitely helps with my anxiety and depression.” |
